# A WHO 2021‐based comprehensive scheme outlining sperm parameters’ associations with IVF outcomes in PGT‐A cycles

**DOI:** 10.1111/andr.13811

**Published:** 2024-11-28

**Authors:** Rossella Mazzilli, Danilo Cimadomo, Federica Innocenti, Marilena Taggi, Greta Chiara Cermisoni, Sara Ginesi, Lisa Dovere, Laura Albricci, Maurizio Guido, Maria Rosaria Campitiello, Susanna Ferrero, Antonio Capalbo, Alberto Vaiarelli, Filippo Maria Ubaldi, Alberto Ferlin, Laura Rienzi, Gianluca Gennarelli

**Affiliations:** ^1^ Department of Clinical and Molecular Medicine University “Sapienza” of Rome Rome Italy; ^2^ IVIRMA Global Research Alliance, Genera, Clinica Valle Giulia Rome Italy; ^3^ Department of Biology and Biotechnology “Lazzaro Spallanzani” University of Pavia Pavia Italy; ^4^ Department of Clinical Medicine, Public Health, Life Sciences and the Environment University of L'Aquila L'Aquila Italy; ^5^ Department of Obstetrics and Gynecology and Physiopathology of Human Reproduction ASL Salerno Salerno Italy; ^6^ Juno Genetics Rome Italy; ^7^ Unit of Molecular Genetics, Center for Advanced Studies and Technology (CAST), “G. d'Annunzio” University of Chieti‐Pescara Chieti Italy; ^8^ Department of Medicine, Unit of andrology and reproductive medicine University of Padova Padova Italy; ^9^ Department of Biomolecular Sciences University of Urbino “Carlo Bo” Urbino Italy; ^10^ IVIRMA Global Research Alliance, Livet Turin Italy; ^11^ Gynecology and Obstetrics, Physiopathology of Reproduction and IVF Unit, Department of Surgical Sciences S. Anna Hospital University of Turin Turin Italy

**Keywords:** cumulative live birth rate, ICSI, paternal age, PGT‐A, sperm factor

## Abstract

**Objective:**

To examine the association between semen parameters, assessed according to World Health Organization (WHO)‐2021 criteria, and paternal body mass index (BMI) and age, with embryological and clinical outcomes in ICSI cycles involving preimplantation genetic testing for aneuploidy (PGT‐A).

**Design:**

Retrospective study at a private in vitro fertilization (IVF) clinic.

**Subjects:**

3101 couples undergoing 4013 intracytoplasmic sperm injection (ICSI) + PGT‐A cycles with own‐oocytes (years 2013–2021).

**Intervention:**

We performed trophectoderm biopsy, and comprehensive chromosome testing to report uniform aneuploidies and vitrified‐warmed euploid single‐blastocyst‐transfers. Regression analyses adjusted for relevant confounders were conducted to outline putative associations of semen analysis and characteristics and paternal BMI and age with all embryological/clinical outcomes.

**Results:**

Maternal age was the only significant confounding variable affecting euploidy blastocyst rate (EBR) (primary embryological outcome). When categorized, motility < 5^th^‐percentile (‐2.5%, 95%CI ‐4.9 to ‐0.2%, *p* = 0.03), concentration plus morphology < 5^th^‐percentile (‐2.7%,95%CI ‐4.8 to ‐0.6%, *p* = 0.01), concentration plus morphology plus motility < 5^th^‐percentile (‐4.0%,95%CI ‐5.5 to ‐2.6%, p < 0.01), obstructive‐azoospermia [OA] (‐5.5%,95%CI ‐9 to ‐2%, *p* = 0.02) and non‐obstructive azoospermia (NOA) (‐5.8%,95%CI ‐10.9 to ‐0.6%, *p* = 0.03) showed significantly lower results compared to all parameters > 5^th^‐percentile. Furthermore, after adjusting for maternal age and the number of metaphase‐II‐oocytes inseminated, the only significant confounding variable affecting the chance of obtaining ≥ 1 live birth among completed cycles (primary clinical outcome) was basal and post sperm processing motility. When categorized, concentration plus morphology plus motility < 5^th^‐percentile (multivariable‐OR: 0.73, 95%CI 0.58–0.93, *p* = 0.01) and OA (multivariable‐OR: 0.47, 95%CI 0.24–0.92, *p* = 0.03) showed significantly lower chances compared to all parameters > 5^th^‐percentile. Advanced paternal age (defined as > 44 years) was associated only with lower day 5‐blastocyst and Gardner's AA‐grade (i.e., top quality) blastocyst rates.

**Conclusions:**

This comprehensive analysis provides IVF professionals with useful figures to counsel infertile couples about their chances of success, taking into account the impact of semen characteristics and paternal BMI and age. These estimates are valuable for personalized decision‐making about the most effective reproductive strategies to adopt, especially not underestimating male factor, by improving sperm concentration and motility whenever possible before assisted reproductive technologies.

## 1 INTRODUCTION

Infertility is defined as the inability to conceive after 12 months of regular unprotected sexual intercourse, a timeframe mostly dependent on maternal age. It affects approximately 10–15% of the couples worldwide, with male factors accounting for 20–70% of the causes of infertility.^1^, ^2^ The introduction of intracytoplasmic sperm injection (ICSI) in 1992 provided an opportunity to treat azoospermic men,^3^ becoming up to date the most commonly used method for fertilization in assisted reproduction technologies (ART) especially to treat severe male factor (SMF). Still, testicular spermatozoa—especially when obtained through testicular sperm extraction (TESE) in patients affected from nonobstructive azoospermia (NOA)—are significantly associated with lower fertilization and blastulation rates,^4^, ^5^, ^6^ perhaps because being less mature and less competent than ejaculated spermatozoa.

There are conflicting data on the effect of male factor infertility on in vitro fertilization (IVF)–ICSI embryological outcomes encompassing blastocyst development and aneuploidy rates.^4^, ^7^, ^8^, ^9^ A previous report from our group suggested that semen quality does not affect euploidy and implantation rates,^4^ and there are two possible explanations for this: (i) impaired early embryo development leading to arrest before blastulation, and/or (ii) oocyte‐derived corrective mechanisms in place before embryonic genome activation.^10^ Similarly, we reported that sex chromosome aneuploidies are independent of both paternal age and semen characteristics.^11^ Regarding paternal body mass index (BMI), although there is evidence that obesity negatively affects semen quality,^12^ data on its impact on ART outcomes are controversial.^13^, ^14^ As for paternal age, then, even if semen quality worsens in older men,^15^ putative effects on embryo developmental competence and aneuploidies are less evident, as also highlighted by a recent meta‐analysis.^16^ On the other hand, “advanced paternal age” could be associated with an increased rate of de novo mutations and epigenetic changes^17^, ^18^ with putative consequences that could be unveiled only through long‐term follow‐up of the babies born. In general, though, a clear consensus on which threshold should be adopted to define “advanced paternal age” is still missing.

An Italian study^19^ investigated the influence of semen quality on cumulative live birth rate (CLBR), suggesting that higher total sperm count values were associated with higher success rates only in case of a limited number of oocytes retrieved; however, azoospermic subjects were excluded. Other than this reference, the CLBR per completed cycle—perhaps the most relevant IVF outcome—is often overlooked in the literature.

Most of the evidences available at present are based on World Health Organization (WHO)‐2010 criteria, which have recently been revised in the WHO‐2021. Interestingly, this sixth edition included data from about 3500 fertile men from 12 countries and five continents, thus validating and expanding the distribution of semen variables of its previous edition. Notably, it was emphasized that—although semen analysis represents the gold standard for male fertility appraisal—the distribution of those data is insufficient to distinguish fertile from subfertile/infertile men yet is very relevant for counseling purposes.

The objective of this study was to evaluate the impact of basic semen analysis parameters, assessed according to WHO‐2021 criteria, as well as paternal BMI and paternal age on embryological and clinical ICSI outcomes among couples undergoing preimplantation genetic testing for aneuploidy (PGT‐A). Our main goal is to provide IVF professionals with information about the impact of these male factors on ICSI outcomes, thus allowing better counseling and informed decision‐making.

## 2 MATERIAL AND METHODS

### 2.1 Participants/materials, setting, and methods

This retrospective single‐center study involved 4013 ICSI cycles with their own oocytes. The study was conducted on 3101 Caucasian couples who requested blastocyst‐stage comprehensive chromosome testing‐based PGT‐A between April 2013 and December 2021. The main embryological outcome was euploid blastocyst rate (EBR) per cohort of inseminated metaphase‐II oocytes. The main clinical outcome was CLBR per complete cycle. A cycle was deemed complete when the couple either delivered or did not deliver and had no euploid blastocyst for transfer.^20^, ^21^ Intermediate embryological and clinical outcomes were also investigated. All parameters were analyzed as continuous variables. For some analyses, paternal age and sperm factor were categorized.

Specifically, the couples were divided into 8 groups according to male partner's sperm parameters based on WHO‐2021 criteria < 5^th^‐percentile plus 2 azoospermic groups: (i) all parameters > 5^th^‐percentile (*N* = 1890, 47.1%), considered as control; (ii) concentration < 5^th^‐percentile (*N* = 149, 3.7%); (iii) motility < 5^th^‐percentile (*N* = 155, 3.9%); (iv) morphology < 5^th^‐percentile (*N* = 405, 10.1%); (v) concentration plus motility < 5^th^‐percentile (N = 70, 1.7%); (vi) concentration plus morphology < 5^th^‐percentile (*N* = 248, 6.2%); (vii) motility plus morphology < 5^th^‐percentile (*N* = 70, 5.0%); (viii) concentration plus motility plus morphology < 5^th^‐percentile (N = 797, 19.9%); (ix) obstructive‐azoospermia (OA) (*N* = 56, 1.4%); (x) NOA (N = 41, 1.0%). The last two conditions were defined after a specific clinical and biological assessment: OA was diagnosed in the presence of normal endocrine function associated with ductal system alteration and/or ejaculatory abnormalities, reduced seminal volume, and sperm recovery in the case of fine‐needle aspiration biopsy (FNA), also confirmed by cytology; the other forms of azoospermia were diagnosed as NOA, also confirmed by histopathology.^10^, ^22^ Regarding ranges of paternal age—as there is no standardized cut‐off to define “advanced paternal age”—the data were clustered following a previously described method as < 38, 38–44 and > 44 years.^23^

The exclusion criteria were gamete donation, PGT for monogenic conditions or abnormal parental karyotypes, NOA not recovering any spermatozoa, NOA caused by genetic conditions, severe female factors (e.g., uterine malformations, severe endometriosis, autoimmune diseases, BMI > 40).

Semen preparation and analysis was carried out at the clinic the same day of ICSI according to WHO guidelines. Briefly, semen was collected from each participant by masturbation after 2–7 days of sexual abstinence. All samples were allowed to liquefy at 37°C, after which macroscopic and microscopic examinations were conducted. The following seminal parameters were assessed: sperm concentration, progressive and total motility, and morphology. Sperm preparation was carried out using either swim‐up methods or, whenever not feasible, sperm washing. FNA and TESE were performed respectively in the case of OA and NOA. If no spermatozoa were retrieved following FNA, TESE was also conducted.^24^, ^25^ Both FNA and TESE were conducted at our clinic. For FNA and TESE, freshly retrieved tissue was washed with a buffered medium (HEPES supplemented with human serum albumin), then disrupted using two glass slides or fine needles to obtain a suspension of small tissue pieces. This suspension was then centrifuged at 300 g  for 8–10 min, and the pellet was inspected under the inverted microscope to assess the presence of sperm.

Recombinant FSH (rec‐FSH) with or without rec‐LH or Human Menopausal Gonadotrophin (HMG) and gonadotropin‐releasing hormone antagonist (GnRH‐ant) ovarian stimulation protocols were conducted in female partners, as previously described.^26^, ^27^ After 35–36 hours from the induction of final oocyte maturation (either GnRH‐agonist or hCG trigger was used), oocyte pick‐up (OPU) was conducted via transvaginal ultrasound‐guided aspiration. ICSI, blastocyst culture, trophectoderm biopsy and vitrification were performed as previously described.^28^, ^29^, ^30^, ^31^ The genetic analysis of trophectoderm biopsies was conducted at an external genetic laboratory with either qPCR or NGS to report uniform aneuploidies.^32^, ^33^, ^34^ Segmental aneuploidies were reported from 2017 onward in a subset of 1991 cycles and 6193 blastocysts.

Each couple provided written informed consent to the treatment. Ethic committee approval was obtained for the retrospective analysis of pseudonymized data aimed at identifying patients’, cycles’ or embryos’ features associated with IVF efficacy and/or efficiency (Rif. 7283, Prot. 0747/2023).

### 2.2 Statistical analyses

Cycles’ data were collected in a relational database (FertilLab). Categorical variables are presented as absolute values, percentages, and 95%CI. Kolmogorov–Smirnov and Shapiro–Wilk tests were conducted to assess the distribution of continuous data, which were then presented as mean with SD and as median with IQR. Fisher's exact test or chi‐squared tests and Kruskal–Wallis or Mann–Whitney *U* tests were used to assess differences between categorical and continuous variables, respectively. Univariate regression analyses were conducted to identify all features associated with all embryological and clinical outcomes under investigation. Putative confounders were identified among maternal age, BMI, FSH, AMH, kind of culture media, kind of incubator, cause of female infertility and duration of couple infertility, kind of comprehensive chromosome testing technology. Univariate analyses were then also conducted to detect associations between semen analysis (volume, concentration, total spermatozoa per ejaculate, basal and post spermatozoa processing motility and morphology), characteristics (ejaculated/FNA/TESE, kind of sperm processing [either swim‐up or, when not possible, sperm washing], fresh/frozen sperm), and paternal BMI and age with all embryological and clinical outcomes under investigation. Multivariate analyses were finally adopted to confirm any putative association. All data were further confirmed with generalized estimated equations to adjust for multiple cycles conducted by the same couple and multiple oocytes/embryos within each cohort. A *p*‐value < 0.05 was considered significant. The software SPSS was used for statistics. Whenever a statistically significant difference was reported in linear and logistic regressions, the software G‐Power 3.1 was used for post‐hoc power calculations.

## 3 RESULTS

### 3.1 Description of the patients’ population

The mean maternal age was 38.9 years and the mean paternal age was 41.9 years, the couples had on average 4 years of infertility, a maternal BMI of 22 and a paternal BMI of 25.6. On average, 10 cumulus‐oocyte‐complexes were retrieved, and 7.3 metaphase‐II oocytes were inseminated. The main causes of female infertility were idiopathic in 3326 cases (83%), endometriosis in 238 (5.9%), endocrine‐ovulatory in 98 (2.4%), tubal factor in 351 (8.7%). The basal characteristics of the patients’ population clustered according to the sperm factor categories based on WHO‐2021 criteria are summarized in Table 1. Women in the control group were generally younger than partners of men characterized by sperm parameters < 5^th^‐percentile or azoospermic.

**TABLE 1:** Basal characteristics according to the sperm factor categories defined based on WHO‐2021 criteria.

Sperm factor categories	Couples (%, *N*)	Maternal age Mean ± SD Median (IQR)	Paternal age Mean ± SD Median (IQR)	Maternal BMI Mean ± SD Median (IQR)	Paternal BMI Mean ± SD Median (IQR)	COCs (*n*.) Mean ± SD Median (IQR)	MII‐oocytes (*n*.) Mean ± SD Median (IQR)
**Total**	(100%, 4013)	38.9 ± 3.3 years 39 (4) years	41.9 ± 5.6 years 41 (7) years	22.0 ± 3.4 kg/m^2^ 21.3 (4) kg/m^2^	25.6 ± 3.2 kg/m^2^ 25 (4) kg/m^2^	10.1 ± 6.3 9 (9)	7.3 ± 4.6 6 (6)
**All sperm parameter > 5^th^ percentile—control**	(47.1%, 1890)	39.3 ± 3.2 years 40 (5) years	41.7 ± 5.4 41 (7) years	21.9 ± 3.3 kg/m^2^ 21.1 (4) kg/m^2^	25.7 ± 3.2 kg/m^2^ 25.1 (4) kg/m^2^	9.4 ± 6.1 8 (8)	6.8 ± 4.4 6 (5)
**Concentration < 5^th^ percentile**	(3.7%, 149)	38.8 ± 3.1 years 39 (4) years^*^	42.1 ± 5.5 years 41 (7) years	22.4 ± 3.5 kg/m^2^ 21.5 (4) kg/m^2^	26.3 ± 3.2 kg/m^2^ 26 (5) kg/m^2^	9.4 ± 5.8 8 (7)	6.9 ± 4.3 6 (5)
**Motility < 5^th^ percentile**	(3.9%, 155)	39.3 ± 3.0 years 40 (5) years	43.3 ± 6.3 years 42 (8) years^**^	22.2 ± 3.6 kg/m^2^ 20.9 (3) kg/m^2^	25.0 ± 3.7 kg/m^2^ 24.9 (4) kg/m^2^	10.2 ± 6.1 9 (10)	7.3 ± 4.5 7 (7)
**Morphology < 5^th^ percentile**	(10.1%, 405)	39.2 ± 3.1 years 40 (5) years	41.8 ± 5.4 years 41 (7) years	21.9 ± 3.4 kg/m^2^ 21 (4) kg/m^2^	25.7 ± 3.2 kg/m^2^ 24.9 (4) kg/m^2^	10.0 ± 6.4 9 (9)	7.4 ± 4.6 6 (6)^*^
**Concentration plus Motility <** **5^th^ percentile**	(1.7%, 70)	38.4 ± 3.5 years 39 (5) years^*^	41.5 ± 5.8 years 41 (7) years	22.2 ± 3.9 kg/m^2^ 21 (4) kg/m^2^	25.9 ± 3.5 kg/m^2^ 25.2 (3) kg/m^2^	10.7 ± 6.5 9 (9)	7.7 ± 4.3 7 (6)^*^
**Concentration plus Morphology <** **5^th^ percentile**	(6.2%, 248)	38.8 ± 3.3 years 39 (4) years^*^	40.8 ± 5.1 years 41 (6) years^*^	21.8 ± 3.1 kg/m^2^ 21 (4) kg/m^2^	25.1 ± 3.3 kg/m^2^ 24.5 (5) kg/m^2^ ^*^	10.5 ± 7.1 10 (9)^*^	7.4 ± 4.6 7 (6)^*^
**Motility plus Morphology <** **5^th^ percentile**	(5.0%, 202)	38.9 ± 3.1 years 39 (4) years	43.2 ± 6.4 years 42 (8.3) years^**^	22.3 ± 3.6 kg/m^2^ 21.5 (4) kg/m^2^	24.6 ± 2.7 kg/m^2^ 24.7 (4) kg/m^2^	10.9 ± 6.2 10 (8)^**^	8.0 ± 4.6 8 (7)^*^
**Concentration plus Motility plus Morphology <** **5^th^ percentile**	(19.9%, 797)	38.3 ± 3.4 years 39 (5) years^*^	41.7 ± 5.6 41 (7)	22.3 ± 3.5 kg/m^2^ 21.8 (4) kg/m^2^ ^*^	25.5 ± 3.1 kg/m^2^ 24.9 (4) kg/m^2^	11.1 ± 6.5 10 (9)^**^	8.0 ± 4.8 7 (7)^**^
**OA**	(1.4%, 56)	38.2 ± 2.3 years 38.5 (5) years^**^	45.3 ± 10.1 years 44 (13.5) years^*^	23.0 ± 3.8 kg/m^2^ 22.6 (5) kg/m^2^	23.3 ± 3.4 kg/m^2^ 23.7 (7) kg/m^2^	11.4 ± 7.0 10 (9)^*^	7.9 ± 4.8 8 (7)^*^
**NOA**	(1.0%, 41)	37.1 ± 3.3 years 38 (4) years^*^	42.6 ± 7.8 years 40 (8.5) years	23.9 ± 5.5 kg/m^2^ 22 (7) kg/m^2^	26.1 ± 2.0 kg/m^2^ 27 (3) kg/m^2^	13.8 ± 2.0 12 (9)^**^	9.9 ± 5.7 9 (8)^**^

*Note*: Mann–Whitney tests were conducted using cycles conducted by men whose sperm parameters were all > 5^th^ percentile as control.

Abbreviations: BMI, body mass index; COCs, cumulus oocyte complexes; MII, metaphase‐II; NOA, on‐obstructive azoospermia; OA, obstructive azoospermia.

* *p* < 0.01 vs. Control.

** *p* < 0.05 vs. Control.

### 3.2 Primary embryological outcome

#### 3.2.1 Associations with euploid blastocyst rate per cohort of inseminated metaphase‐II oocytes

Paternal age and BMI did not show any association with the EBR per inseminated metaphase‐II oocytes. Maternal age was the only confounder on this outcome. All analyses were therefore adjusted accordingly. When tested as a continuous variable, the only semen analysis parameters associated with this outcome were basal and post spermatozoa processing motility (unstandardized coefficient B +0.1%, *p* < 0.001 for both). When categorizing the sperm factor, instead, only concentration plus morphology < 5^th^ percentile (unstandardized coefficient B ‐2.7%, 95%CI ‐4.8 to ‐0.6%, *p* = 0.01), concentration plus motility plus morphology < 5^th^ percentile (unstandardized coefficient B ‐4.0%, 95%CI ‐5.5 to ‐2.6%, *p* < 0.01), OA (unstandardized coefficient B ‐5.5%, 95%CI ‐9 to ‐2%, *p *= 0.02) and NOA (unstandardized coefficient B ‐5.8%, 95%CI ‐10.9 to ‐0.6%, *p* = 0.03) showed significantly lower EBR than the control (Table 2).

**TABLE 2:** Embryological outcomes according to the spermatozoa factor categories defined based on WHO‐2021 criteria.

Sperm factor categories	Fertilization rate per cohort of MII‐oocytes	Blastulation rate per cohort of 2PN‐zygotes	Euploidy rate per cohort of biopsied blastocysts	EBR per cohort of MII‐oocytes
**All sperm parameter > 5^th^ percentile—control** Mean ± SD Median (IQR)	**76.3 ± 22.9%** **80% (33%)**	**49.4 ± 30.8%** **50% (46%)**	**36.3 ± 36.3%** **33% (67%)**	**14.5% ± 20.1%** **6% (25%)**
**Concentration < 5^th^ percentile** *Unstandardized coefficient B, 95% CI, p‐value*	−0.9%, from ‐4.6 to +2.9%, *p* = 0.64	−6.1%, from ‐11 to ‐1.2%, *p* = 0.02^*^ Power < 60%	+1.4%, from ‐4.9% to +7.7%, *p* = 0.66^**^	−0.5%, from ‐4% to +3.1%, *p* = 0.80^***^
**Motility < 5^th^ percentile** *Unstandardized coefficient B, 95% CI, p‐value*	−0.5%, from ‐4.1 to +3.1%, *p* = 0.77 °	−2.7%, from ‐7.2 to +1.8%, *p* = 0.23^*^	−0.6%, from ‐6.4 to +5.3%, *p* = 0.85^**^	−2.5%, from ‐4.9 to ‐0.2%, *p* = 0.03^***^ Power = 99%
**Morphology < 5^th^ percentile** *Unstandardized coefficient B, 95% CI, p‐value*	−2.7%, from ‐5.2 to ‐0.2%, *p* = 0.05 Power < 60%	+1.8%, from ‐1.5 to +5.1%, *p* = 0.26^*^	−1.0%, from ‐4.7 to +2.6%, *p* = 0.58^**^	−0.9%, from ‐2.7 to +1.0%, *p* = 0.35^***^
**Concentration plus Motility <** **5^th^ percentile** *Unstandardized coefficient B, 95% CI, p‐value*	+1.2%, from ‐4.2 to +6.7%, *p* = 0.66 °	−3.0%, from ‐9.4 to +3.3%, *p* = 0.34^*^	+0.5%, from ‐8.2 to +9.1%, *p* = 0.92^**^	−2.2%, from ‐5.6 to +1.1%, *p* = 0.19^***^
**Concentration plus Morphology <** **5^th^ percentile** *Unstandardized coefficient B, 95% CI, p‐value*	−2.8%, from ‐5.7 to +0.1%, *p* = 0.06	−3.6%, from ‐7.4 to +0.1%, *p* = 0.06^*^	−0.7%, from ‐5.5 to +4.1%, *p* = 0.78^**^	−2.7%, from ‐4.8 to ‐0.6%, *p* = 0.01^***^ Power = 99%
**Motility plus Morphology <** **5^th^ percentile** *Unstandardized coefficient B, 95% CI, p‐value*	+0.6%, from ‐2.6 to +3.8%, *p* = 0.70	−1.1%, from ‐5.5 to +3.3%, *p* = 0.65^*^	−1.1%, from ‐6.2 to +4.1%, *p* = 0.69^**^	−1.2%, from ‐3.7 to +1.3%, *p* = 0.34^***^
**Concentration plus Motility plus Morphology <** **5^th^ percentile** *Unstandardized coefficient B, 95% CI, p‐value*	−2.6%, from ‐4.9 to ‐0.3%, *p* = 0.02 ° Power = 99%	−7.1%, from ‐9.7 to ‐4.4%, *p* < 0.01^*^ Power = 99%	−0.8%, from ‐3.9 to +2.4%, *p* = 0.63^**^	−4.0%, from ‐5.5 to ‐2.6%, *p* < 0.01^***^ Power = 99%
**OA** *Unstandardized coefficient B, 95% CI, p‐value*	−10.3%, from ‐16.1 to ‐4.6%, *p* < 0.01 Power = 93%	−9.4%, from ‐17.5 to ‐1.3%, *p* = 0.02^*^ Power < 60%	+4.1%, from ‐6.8 to +14.9%, *p* = 0.46^**^	−5.5%, from ‐9 to ‐2%, *p* = 0.02^***^ Power < 60%
**NOA** *Unstandardized coefficient B, 95% CI, p‐value*	−16.2%, from ‐24.1 to ‐8.4%, *p* < 0.01 Power = 99%	−7%, from ‐15.5 to +1.4%, *p* = 0.10^*^	+6.1%, from ‐6.5 to +18.8%, *p* = 0.34^**^	−5.8%, from ‐10.9 to ‐0.6%, *p* = 0.03^***^ Power < 60%

*Note*: All data and *p*‐values were analyzed with generalized estimated equations to adjust for multiple cycles conducted by the same couple.

Abbreviations: EBR, euploid blastocyst rate; MII, metaphase‐II; NOA, non‐obstructive azoospermia; OA, obstructive azoospermia; PN, pronuclei.

adjusted for the sperm preparation method used;

* adjusted for maternal age and the kind of culture media used;

** adjusted for maternal age and the kind of comprehensive chromosome testing technology used;

*** adjusted for maternal age.

### 3.3 Primary clinical outcomes

#### 3.3.1 Associations with the chance to obtain at least one fertilized oocyte, blastocyst, euploid blastocyst and of cumulative live birth per conclude cycle

Paternal age and BMI did not show any association with the chance to obtain at least one fertilized oocyte, at least one blastocyst, at least one euploid blastocyst and with the CLBR per completed cycle. The number of inseminated metaphase‐II oocytes was instead associated with all these outcomes. This variable was therefore adopted to adjust all investigations. Maternal age was also found to be significantly associated with the chance to obtain at least one blastocyst, at least one euploid blastocyst and with the CLBR per completed cycle. Thus, this feature was adopted to adjust these investigations along with the number of inseminated metaphase‐II oocytes.

When tested as continuous variables, the only semen analysis parameters associated with all these outcomes were basal (multivariable‐ORs for its association with the chance to obtain ≥1 fertilized oocyte, ≥1 blastocyst, ≥1 euploid and ≥1 LB among completed cycles: 1.016, 1.015, 1.011 and 1.009 always with a *p* < 0.001) and post sperm processing motility (multivariable‐ORs for its association with the chance to obtain ≥1 fertilized oocyte, ≥1 blastocyst, ≥1 euploid and ≥1 LB among completed cycles: 1.015, 1.013, 1.012 and 1.012 always with a *p* < 0.001). When categorizing semen analysis based on WHO‐2021, instead, only concentration plus motility plus morphology < 5^th^ percentile was significantly associated with lower chances for all cycle outcomes and in particular with a multivariable‐OR for the CLBR per completed cycle of 0.73, 95%CI 0.58 ‐ 0.93, *p* = 0.01 versus the control. Among OA, a lower chance to obtain ≥1 blastocyst was reported and a lower CLBR per completed cycle of 0.47, 95%CI 0.24–0.92, *p* = 0.03. Among NOA, instead, only a lower chance to obtain ≥1 blastocyst was reported (Table 3).

**TABLE 3:** Chance of obtaining at least one fertilized oocyte, blastocyst, euploid blastocyst, and of cumulative live birth rate per concluded cycle according to sperm factor analysis based on WHO‐2021 criteria.

Sperm factor categories	Chance of obtaining at least one fertilized oocyte	Chance of obtaining at least one blastocyst	Chance of obtaining at least one euploid blastocyst	Cumulative live birth rate per concluded cycle
**All sperm parameter > 5^th^ percentile—control *N, %***	***N* = 1835/1890, 97.1%**	***N* = 1590/1890, 84.1%**	***N* = 955/1890, 50.5%**	***N* = 460/1727, 26.6%** **Cycles still open: 163**
**Concentration < 5^th^ percentile** *Multivariate OR, 95%CI, p‐value*	1.48 95%CI 0.42–5.18, *p* = 0.54	0.66 95%CI 0.40–1.08, *p* = 0.10	0.76 95%CI 0.51–1.13, *p* = 0.17	0.82 95%CI 0.51–1.32, *p* = 0.42 Cycles still open: 15
**Motility < 5^th^ percentile** *Multivariate OR, 95%CI, p‐value*	1.07 95%CI 0.35–3.25, *p* = 0.91	1.45 95%CI 0.86–2.46, *p* = 0.17	0.84 95%CI 0.59–1.22, *p* = 0.37	0.70 95%CI 0.44–1.10, *p* = 0.12 Cycles still open: 10
**Morphology < 5^th^ percentile** *Multivariate OR, 95%CI, p‐value*	0.76 95%CI 0.40–1.43, *p* = 0.39	0.89 95%CI 0.64–1.24, *p* = 0.49	1.09 95%CI 0.83–1.42, *p* = 0.54	1.02 95%CI 0.76–1.38, *p* = 0.88 Cycles still open: 34
**Concentration plus Motility <** **5^th^ percentile** *Multivariate OR, 95%CI, p‐value*	0.72 95%CI 0.08 – 6.26, *p* = 0.77	1.23 95%CI 0.53–2.83, *p* = 0.63	1.20 95%CI 0.67–2.18, *p* = 0.54	0.92 95%CI 0.52–1.65, *p* = 0.79 Cycles still open: 7
**Concentration plus Morphology <** **5^th^ percentile** *Multivariate OR, 95%CI, p‐value*	0.92 95%CI 0.40–2.10, *p* = 0.84	0.71 95%CI 0.48–1.05, *p* = 0.09	1.85 95%CI 0.61–1.18, *p* = 0.85	0.73 95%CI 0.50–1.07, *p* = 0.11 Cycles still open: 23
**Motility plus Morphology <** **5^th^ percentile** *Multivariate OR, 95%CI, p‐value*	1.69 95%CI 0.49–5.86, *p* = 0.41	0.94 95%CI 0.59–1.50, *p* = 0.80	0.91 95%CI 0.64–1.28, *p* = 0.59	0.98 95%CI 0.66–1.45, *p* = 0.92 Cycles still open: 23
**Concentration plus Motility plus Morphology <** **5^th^ percentile** *Multivariate OR, 95%CI, p‐value*	0.42 95%CI 0.25–0.72, *p* < 0.01 Power = 99%	0.53 95%CI 0.42–0.68, *p* < 0.01 Power = 99%	0.75 95%CI 0.60–0.92, *p* < 0.01 Power = 91%	0.73 95%CI 0.58–0.93, *p* = 0.01 Cycles still open: 75 Power = 90%
**OA** *Multivariate OR, 95%CI, p‐value*	0.66 95%CI 0.13–3.34, *p* = 0.61	0.29 95%CI 0.12–0.67, *p* < 0.01 Power = 99%	0.72 95%CI 0.33–1.54, *p* = 0.39	0.47 95%CI 0.24–0.92, *p* = 0.03 Cycles still open: 5 Power < 60%
**NOA** *Multivariate OR, 95%CI, p‐value*	0.42 95%CI 0.04–4.27, *p* = 0.46	0.38 95%CI 0.15–1.00, *p* = 0.05 Power = 77%	0.81 95%CI 0.33 – 2.04, *p* = 0.66	0.90 95%CI 0.28–2.90, *p* = 0.86 Cycles still open: 9

*Note*: All data were adjusted for maternal age and number of inseminated MII oocytes, except for the chance to obtain at least one fertilized oocyte for which maternal age was not a confounder.

Generalized estimated equations were conducted to adjust for multiple cycles conducted by the same couple.

Abbreviations: OA, obstructive azoospermia; NOA, non‐obstructive azoospermia.

### 3.4 Secondary embryological and clinical outcomes

#### 3.4.1 Associations with fertilization rate per cohort of inseminated metaphase‐II oocytes

Only the sperm processing method was identified as a confounder on the fertilization rate per cohort of inseminated metaphase‐II oocytes. Specifically, sperm washing was conducted in 4.5% of cycles (*N* = 7/155) where sperm motility was < 5^th^‐percentile, 11.4% of cycles (*N* = 8/70) where both concentration and motility were < 5^th^‐percentile, 5.4% of the cycles (*N *= 11/202) where both motility and morphology were < 5^th^‐percentile, 29.5% of the cases where all sperm parameters were < 5^th^‐percentile, and all surgical sperm retrieval procedures—was associated with poorer fertilization, thus it was used to adjust the investigation. Paternal age and BMI showed no association, instead. When tested as continuous variables, the only semen analysis parameters associated with this intermediate embryological outcome were basal and post sperm processing motility (unstandardized coefficient B +0.1%, *p* < 0.001 for both). When categorizing the sperm factor, instead, only men with morphology < 5^th^ percentile (unstandardized coefficient B ‐2.7%, 95%CI ‐5.2 to ‐0.2%, *p* = 0.03), concentration plus motility plus morphology < 5^th^ percentile (unstandardized coefficient B ‐2.6%, 95%CI ‐4.9 to ‐0.3%, *p* = 0.02), OA (unstandardized coefficient B ‐10.3%, 95%CI ‐16.1 to ‐4.6%, p < 0.01) and NOA (unstandardized coefficient B ‐16.2%, 95%CI ‐24.1 to ‐8.4%, p < 0.01) showed significantly lower fertilization rates than the control (Table 2).

#### 3.4.2 Associations with atypically pronucleated zygotes

Among all zygotes (excluding non‐fertilized oocytes and ≥ 4 pronucleated (PN) zygotes), the distribution of atypically pronucleated zygotes (showing a single pronucleus [1PN], three pronuclei [3PN], and two large plus 1 small pronuclei [2.1PN])^35^ was comparable across all groups (Supporting Figure 1), nor any other paternal characteristic showed an association with this outcome.

#### 3.4.3 Associations with blastulation rate per cohort of 2PN zygotes

Paternal age and BMI did not show any association with the blastulation rate per cohort of 2PN zygotes. Maternal age and kind of culture media were instead reported as relevant confounders. All analyses were therefore adjusted accordingly. When tested as a continuous variable, the only semen analysis parameters associated with this outcome were basal and sperm processing motility (unstandardized coefficient B +0.2%, p < 0.001 and +0.1%, *p* < 0.001, respectively), and basal sperm concentration (unstandardized coefficient B +0.1%, *p* < 0.001). When categorizing the sperm factor, instead, only men with concentration < 5^th^ percentile (unstandardized coefficient B ‐6.1%, 95%CI ‐11.2 to ‐1.2%, *p* = 0.02), concentration plus motility plus morphology < 5^th^ percentile (unstandardized coefficient B ‐7.1%, 95%CI ‐9.7 to ‐4.7%, p < 0.01) and OA (unstandardized coefficient B ‐9.4%, 95%CI ‐17.5 to ‐1.3%, p = 0.02) showed significantly lower blastulation rates than the control (Table 2).

#### 3.4.4 Blastocyst day of development and morphological quality

Only categorized paternal age showed an association with day 5 blastocyst and Gardner's AA‐grade blastocyst^36^ rates among men > 44 versus men < 38 years, which was significant even when adjusted for maternal age (*N* = 919/3127, 29.4% versus *N* = 1131/3308, 34.2%; multivariable‐OR: 0.86, 95%CI 0.74–0.99, *p* = 0.04; *N* = 1387/3127, 44.4% versus *N* = 1732/3308, 52.4%; multivariable‐OR: 0.89, 95%CI 0.8‐0.99, *p* = 0.05) (Supporting Figure 2). Semen analysis parameters, instead, either continuous or categorized according to WHO‐2021, did not show any association with these outcomes.

#### 3.4.5 Associations with euploidy rate per cohort of biopsied blastocysts

Paternal age and BMI did not show any association with the euploidy rate per cohort of biopsied blastocysts. Maternal age and the kind of comprehensive chromosome testing technique used (either qPCR or NGS) were the only confounders identified, and all analyses were adjusted accordingly. All semen analysis parameters and sperm factor categories were not associated with this outcome (Table 2). In a sub‐analysis adjusted for maternal age and blastocyst's quality, no association was reported either with the prevalence of segmental aneuploidies (Supporting Table 1).

#### 3.4.6 Associations with the LBR per first vitrified‐warmed single euploid blastocyst transfer

**FIGURE 1:**
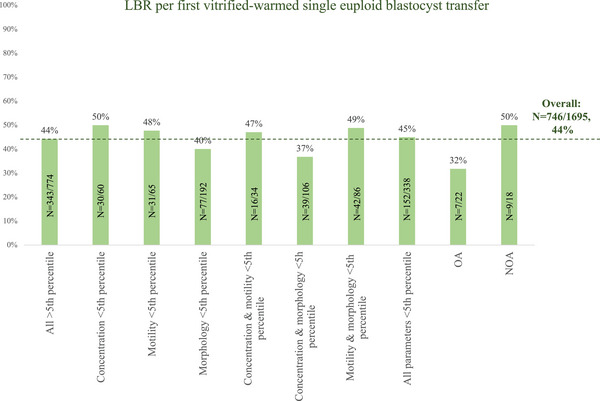
Live birth rate (LBR) per first vitrified‐warmed single euploid blastocyst transfer according to semen analysis based on WHO‐2021. No significant difference was reported versus the control, even when adjusting for day of biopsy and blastocyst's morphological quality.

The LBR per first vitrified‐warmed single euploid blastocyst transfer was adjusted for morphological quality, and day of development (i.e., the only significant confounders). No association was shown with paternal age and BMI, nor with the sperm parameters continuous or categorized according to WHO‐2021 criteria (Figure 1).

#### 3.4.7 Effect of the different sperm factor categorization based on WHO‐2021 versus WHO‐2010

The transition from the 2010 to the 2021 version of the WHO involved 87.4% of the patients staying in the same sperm factor category, while 12.6% changing it. Specifically, with respect to WHO‐2010 categories, 8.3% of the normozoospermic, 21% of the oligozoospermic, 8% of the asthenozoospermic, 27% of the teratozoospermic, 36.1% of the oligoteratozoospermic, and 20% of the asthenoteratozoospermic patients changed their category to a worse category based on sperm characteristics < 5^th^‐percentile (Supporting Table 2). The reanalysis of the main outcomes of the present study based on WHO‐2010 categorization outlined superimposable results (Supporting Table 3).

## 4 DISCUSSION

This study comprehensively evaluated the impact of semen analysis and characteristics, paternal BMI and age on all embryological and clinical outcomes during PGT‐A cycles conducted at the blastocyst stage. It is an updated and improved version of a previous manuscript from our group,^4^ as it is based on WHO‐2021 criteria, encompassed a larger population, included more detailed analyses (i.e., also atypically pronucleated zygotes and segmental aneuploidies were reported), and investigated OA and NOA cycles only in case both surgical retrieval and ICSI+PGT treatment were conducted at our clinic.

The impact of maternal age and ovarian reserve (evaluated through the number of metaphase II oocytes retrieved and inseminated) on embryological and clinical outcomes was confirmed more relevant than the impact of the male factor. Specifically, the former impact largely dilutes the immediate consequence of the latter. However, if the concentration, motility, and morphology are all < 5^th^‐percentile, all outcomes showed a significant decrease with high statistical power. The only exceptions were the EBR per cohort of biopsied blastocysts—an outcome mostly dependent on maternal meiotic missegregations^37^—and the LBR per first vitrified‐warmed single euploid blastocyst transfer, whose confounding factors still remain a “black box”.^38^

Reduced basal and post sperm processing motility and, from a broader perspective, patients with concentration plus morphology plus motility < 5^th^‐percentile or azoospermic, were the characteristics associated with worse embryological outcomes and CLBR per completed PGT‐A cycle. Moreover, among all semen analysis characteristics, the semen processing protocol was reported the only feature found to be associated with worse outcomes; specifically, lower fertilization rates were reported among cycles where the sperm washing protocol was adopted. These findings all together highlight the importance of sperm motility, evidence also supported by previous data produced via ICSI on donor oocytes.^39^ In this regard, sperm chromatin and mitochondrial integrity, both critical for fertilization and further developmental competence, result in more motile sperm capable of swimming up the female genital tract, penetrating the oocyte and forming the male pronucleus. Benefits that seem to apply also to ART.^40^, ^41^

Advanced paternal age (here categorized as > 44 years) was reported to be associated with slower blastocyst development and poorer blastocyst morphological quality. While these two variables are indeed associated—even in the context of euploid blastocyst transfers^38^—with reduced reproductive competence, slower growing blastocysts and/or blastocysts of a low morphological quality still do implant at reasonably good rates. Indeed, no significant impact of advanced paternal age was reported on any clinical outcomes or on euploidy rates, evidence that aligns with a recent meta‐analysis.^16^ Again, a putative paternal age effect might have been diluted by maternal age, as these two variables are highly associated. Paternal BMI also showed no association with all outcomes under investigation, another evidence in line with a recent detailed investigation.^14^

Interestingly, semen analysis and characteristics, paternal BMI and age did not report any effect on atypically pronucleated zygotes (1PN or 3PN rates), confirming the results from another investigation on 1855 ICSI cycles.^42^

When our IVF center switched the comprehensive chromosome testing technology adopted for PGT‐A purposes from qPCR to NGS, uniform segmental aneuploidies were started to be reported^34^ (alleged mosaicism has never been reported instead, a decision which was confirmed clinically sound in a previous investigation^43^). This change did not show any association with the primary outcome of this study, namely EBR per cohort of inseminated oocytes (unstandardized coefficient B: ‐1.3%, 95%CI ‐2.6% to +0.1%, *p* = 0.1), nor with the CLBR per cycle (multivariable‐OR = 1.09, 95%CI 0.92–1.3, p = 0.3). The only effect was a lower euploidy rate per cohort of biopsied blastocysts with NGS, which was predictable and imputable to the prevalence of blastocysts exclusively affected from uniform segmental aneuploidies. Specifically, the mean euploidy rate per cohort of biopsied blastocysts when qPCR was used was 39.6%, while it decreased to 36.5% when NGS was used with an average 2.9% rate of blastocysts carrying exclusively segmental aneuploidies. In other terms, if segmental aneuploidies would have not been reported by NGS in this period (from 2017 onward), the mean euploidy rate per cohort of biopsied blastocysts would have been 39.4%, namely superimposable to what was achieved when qPCR was adopted (2014–2017). Based on this, the absence of associations for the variables under investigation with the euploidy rate per cohort of biopsied blastocysts was therefore confirmed by adjusting for the kind of comprehensive chromosome testing technology along with maternal age as key confounders.

In the subset of our data where segmental aneuploidies were detected by NGS, their overall prevalence (i.e., also in association with other full chromosome uniform aneuploidies) was independent from semen characteristics. This finding has been confirmed by some studies, but in contrast with others. For instance, a study conducted on a large cohort of embryos derived from young oocyte donors with available PGT‐A data, showed an increase of segmental aneuploidies with paternal age, mainly in subjects older than 50.^44^ Similarly, it has also been reported that segmental aneuploidies are more likely to be of paternal origin than whole chromosome aneuploidies,^45^ although this association did not reach statistical significance. Therefore, the mechanisms involved in segmental aneuploidies origin might be different from whole chromosome aneuploidies, an aspect that deserves further investigation in the future.

Regarding newborn follow‐up data, advanced paternal age has been associated with a higher risk of de novo single gene defects, whose effect might be visible much later in life.^46^, ^47^, ^48^ In fact, it has been reported that approximately 80% of all de novo mutations are paternally derived and paternal age at conception influences also their number in the offspring, mainly as a function of the number of mitotic divisions, and thus genome replications, in the life of a spermatozoa.^49^ The mechanisms at the roots of this could be errors in DNA replication, ineffective repair of DNA damage, as well as prolonged exposure to mutagens throughout life.^47^ Data on this aspect are missing from our study, therefore we advocate for future dedicated investigations.

Finally, we investigated the effect of the different sperm factor categorization based on WHO‐2021 versus WHO‐2010. The last edition of WHO analyzed 3,500 fertile men from 12 countries and 5 continents highlighting all sperm parameters (concentration, motility and morphology) below the 5th‐percentile. While in the WHO‐2010 these thresholds were used to outline an issue, in the WHO‐2021 they were suggested only as information to be discussed with the patients to guide further investigations or treatment options. Interestingly, although the distribution of the patients in the different categories would vary from WHO‐2010 to WHO‐2021, all statistically significant impacts outlined in the present study were confirmed. This supports the validity also of previous analyses on this topic conducted according to WHO‐2010 categorization.

### 4.1 Limitations

A limitation of the present study is the lack of data on sperm DNA fragmentation, which has been suggested as a putative predictor of poorer IVF outcomes,^8^, ^50^ but that is not routinely performed at our center for all couples, as suggested by the guidelines of two relevant Italian scientific societies in this field.^22^ Other limitations of this study are that some confounders—although being controlled for in linear and logistic regressions—were unbalanced among the study groups categories, and that 9% of the cycles were not completed at the time of manuscript drafting. Furthermore, the sample size in the groups of surgically retrieved spermatozoa is still low. Indeed, azoospermic men, both OA and NOA, represent just a minority of the study population (1.4% and 1.0%, respectively), in turn reducing the power of some of our analyses. Also, these men are generally partners of younger women with better prognosis than the general population, which may compensate for their reduced fertility.^51^ In addition, NOA patients because of genetic causes were excluded from this study, thus deserving specific appraisal. Of note, the study is based only on Caucasian couples undergoing PGT‐A and should be extended to other ethnicities. At last, it could be interesting to conduct further studies also based on different classification methods, such as the Hamilton's total motile sperm count.^52^ Of note, these results should be extended to include the management—in terms of both diagnosis and specific therapies—of male partners before ICSI. This will allow us to determine the causes of any impact observed, namely whether this impact is due to a direct effect of the seminal parameters themselves, or if the origin of those seminal alterations is responsible for poorer outcomes.

### 4.2 Conclusions

By comprehensively accounting for semen analysis and characteristics and paternal BMI and age, this report provides reproductive professionals with useful information to counsel infertile couples about their expected outcomes and chance of success during IVF, updated on the basis of WHO‐2021. These estimates are valuable for personalized decision‐making in IVF regarding the most effective clinical strategies to apply, especially not to underestimate the influence of the sperm characteristics and to counsel about the IVF outcome accordingly. In view of the evidence summarized in this study, it is advisable to attempt to improve especially sperm concentration and motility, whenever feasible before IVF. Specific studies in this regard are warranted.

## AUTHOR CONTRIBUTIONS

Rossella Mazzilli and Danilo Cimadomo designed the study. Federica Innocenti, Lisa Dovere, Sara Ginesi, Laura Albricci, Susanna Ferrero, and Alberto Vaiarelli collected the data. Danilo Cimadomo analyzed the data. Rossella Mazzilli, Danilo Cimadomo, and Sara Ginesi drafted the manuscript. All authors contributed to the discussion of the results.

## CONFLICT OF INTEREST STATEMENT

The authors declare no conflicts of interest.

## FUNDING INFORMATION

The authors declare no financial interest related to the content of this study.

## Supporting information



Supporting information

## Data Availability

The data that support the findings of this study are available from the corresponding author upon reasonable request.
