# Supplementary material for: A WHO 2021‐based comprehensive scheme outlining sperm parameters’ associations with IVF outcomes in PGT‐A cycles
Source: Andrology. 2024 Nov 28;13(7):1745–56. doi: 10.1111/andr.13811 (PMC12476224; doi:10.1111/andr.13811)
Supplement: Supplementary file 1 — Supporting information [file ANDR-13-1745-s001.docx]

**Supplemental Table 1.** Prevalence of blastocysts with segmental aneuploidies according to sperm factor as defined based on WHO-2021 criteria.

p-value are adjusted for maternal age, blastocyst’s quality and multiple blastocysts from the same cohort through generalized estimated equations.

*OA, obstructive azoospermia; NOA, non-obstructive azoospermia*

|  | **Prevalence of blastocysts with segmental aneuploidies**  N (%) |
| --- | --- |
| **Versus sperm analysis based on WHO-2021** | |
| **All sperm parameter > 5^th^ percentile - control** | N=172/3026 (5.7%), - |
| **Concentration < 5^th^ percentile** | N=17/285 (6.0%), p=0.4 |
| **Motility < 5^th^ percentile** | N=15/245 (6.1%), p=0.4 |
| **Morphology < 5^th^ percentile** | N=16/505 (3.2%), p=0.2 |
| **Concentration plus Motility < 5^th^ percentile** | N=8/155 (5.2%), p=0.9 |
| **Concentration plus Morphology < 5^th^ percentile** | N=17/342 (5.0%), p=0.6 |
| **Motility plus Morphology < 5^th^ percentile** | N=16/280 (5.7%), p=0.8 |
| **Concentration plus Motility plus Morphology < 5^th^ percentile** | N=86/1211 (7.1%), p=0.1 |
| **OA** | N=3/84 (3.6%), p=0.7 |
| **NOA** | N=2/60 (3.3%), p=0.8 |
| **Versus paternal age range** | |
| **<38 years - control** | N=132/2135 (6.2%), - |
| **38-44 years** | N=114/2125 (5.4%), p=0.8 |
| **>44 years** | N=106/1933 (5.5%), p=0.6 |

**Supplemental Table 2. Differences between the categorization defined by WHO-2021 versus WHO-2010 on the population of patients in this study.**

|  |  | **WHO 2021** | | | | | | | | |
| --- | --- | --- | --- | --- | --- | --- | --- | --- | --- | --- |
|  |  | **All parameters >5^th^ perc** | **Conc < 5^th^ perc** | **Mot < 5^th^ perc** | **Morph < 5^th^ perc** | **Conc plus Mot < 5^th^ perc** | **Conc plus Morph < 5^th^ perc** | **Mot plus Morph < 5^th^ perc** | **Conc plus Mot plus Morph < 5^th^ perc** | **TOT** |
| **WHO 2010** | **N**  *% within N*  *% overall* | 1890  91.7%  48.3% | 51  2.5%  1.3% | 111  5.4%  2.8% | 0  -  - | 8  0.4%  0.2% | 0  -  - | 0  -  - | 0  -  - | 2060 |
|  | **O**  *% within O*  *% overall* | -  -  - | 98  79%  2.5% | 0  -  - | 0  -  - | 26  21%  0.7% | 0  -  - | 0  -  - | 0  -  - | 124 |
|  | **A**  *% within A*  *% overall* | -  -  - | -  -  - | 44  92%  1.1% | 0  -  - | 4  8%  0.1% | 0  -  - | 0  -  - | 0  -  - | 48 |
|  | **T**  *% within T*  *% overall* | -  -  - | -  -  - | -  -  - | 405  73%  10.3% | 0  -  - | 44  8%  1.1% | 93  17%  2.4% | 15  3%  0.4% | 557 |
|  | **OA**  *% within OA*  *% overall* | -  -  - | -  -  - | -  -  - | -  -  - | 32  100%  0.8% | 0  -  - | 0  -  - | 0  -  - | 32 |
|  | **OT**  *% within OT*  *% overall* | -  -  - | -  -  - | -  -  - | -  -  - | -  -  - | 204  63.9%  5.2% | 0  -  - | 115  36.1%  2.9% | 319 |
|  | **AT**  *% within AT*  *% overall* | -  -  - | -  -  - | -  -  - | -  -  - | -  -  - | -  -  - | 109  80%  2.8% | 28  20%  0.7% | 137 |
|  | **OAT**  *% within OAT*  *% overall* | -  -  - | -  -  - | -  -  - | -  -  - | -  -  - | -  -  - | -  -  - | 639  100%  16.3% | 639 |
|  | **TOT** | 1890 | 149 | 155 | 405 | 70 | 248 | 202 | 797 | **3916** |

**Supplemental Table 3. Main study outcomes according to WHO-2010 categories.**

^#^ Euploid blastocyst rate (EBR) was adjusted for maternal age, ^*^ cumulative live birth rate (cLBR) was adjusted for maternal age and number of inseminated MII oocytes.

Generalized estimated equations were conducted to adjust for multiple cycles conducted by the same couple.

| **Sperm factor WHO 2010 categories** | **EBR per cohort of MII-oocytes**  Mean ± SD  Media (IQR) | **Cumulative live birth rate per concluded cycle** |
| --- | --- | --- |
| **N** | **14.3% ± 19.9%**  **5% (25%)** | **N=498/1884, 26.4%**  **Cycles still open: 176** |
| **O**  *Unstandardized coefficient B, 95% CI, p-value* | -1.1%,  from -4.6% to +2.4%, p=0.55 ^#^ | 0.77  95%CI 0.46 - 1.28, p=0.32 *  Cycles still open: 14 |
| **A**  *Unstandardized coefficient B, 95% CI, p-value* | +1.0%,  from -3.8 to +5.8%, p=0.68 ^#^ | 0.76  95%CI 0.38 - 1.52, p=0.43 *  Cycles still open: 2 |
| **T**  *Unstandardized coefficient B, 95% CI, p-value* | -0.5%,  from -2 to +1.1%, p=0.58^#^ | 1.06  95%CI 0.82 - 1.38, p=0.64 *  Cycles still open: 49 |
| **OA**  *Unstandardized coefficient B, 95% CI, p-value* | -0.9%,  from -6.7 to +5%, p=0.77 ^#^ | 0.86  95%CI 0.4 – 1.8, p=0.69 *  Cycles still open: 3 |
| **OT**  *Unstandardized coefficient B, 95% CI, p-value* | -2.5%,  from -4.6 to -0.5%, p=0.02 ^#^  Power=99% | 0.78  95%CI 0.55 - 1.09, p=0.15 *  Cycles still open: 29 |
| **AT**  *Unstandardized coefficient B, 95% CI, p-value* | -2.2%,  from -4.9 to +0.5%, p=0.10 ^#^ | 0.88  95%CI 0.55 – 1.42, p=0.59 *  Cycles still open: 14 |
| **OAT**  *Unstandardized coefficient B, 95% CI, p-value* | -4.4%,  from -5.9 to -2.9%, p<0.01 ^#^  Power=99% | 0.69  95%CI 0.53 - 0.89, p=0.005 *  Cycles still open: 63  Power=95% |
| **OA**  *Unstandardized coefficient B, 95% CI, p-value* | -5.2%,  from -8.7 to -1.8%, p<0.01 ^#^  Power<60% | 0.49  95%CI 0.25 – 0.94, p=0.033 *  Cycles still open: 5  Power=53% |
| **NOA**  *Unstandardized coefficient B, 95% CI, p-value* | -5.5%,  from -10.7 to -0.4%, p=0.04 ^#^  Power<60% | 0.92  95%CI 0.29 – 2.9, p=0.88 *  Cycles still open: 9 |
